# A novel curriculum to train physician assistant students how to write effective discharge summaries

**DOI:** 10.1080/10872981.2019.1648944

**Published:** 2019-08-01

**Authors:** Kahli E. Zietlow, Megan Gillum, Sarah L. Hale, April Stouder, Melinda Blazar, Nicholas M Hudak, David Ming

**Affiliations:** a Department of Medicine, Division of Geriatrics, Duke University Medical Center, Durham, NC, USA; b Department of Medicine, Division of General Internal Medicine, Hospital Medicine Programs, DUMC, Durham, NC, USA; c Department of Biostatistics and Bioinformatics, DUMC, Durham, NC, USA; d Duke Physician Assistant Program, DUMC, Durham, NC, USA; e Department of Pediatrics, Division of Pediatric Hospital Medicine, DUMC, Durham, NC, USA

**Keywords:** Discharge summaries, curriculum development, physician assistants, feedback, transitions of care

## Abstract

**Background**: Physician assistants (PAs) are an integral part of inpatient care teams, but many PAs do not receive formal education on authoring discharge summaries. High-quality discharge summaries can mitigate patient risk during transitions of care by improving inter-provider communication.

**Objective**: To understand the current state of discharge summary education at our institution, and describe a novel curriculum to teach PA students to write effective discharge summaries.

**Design**: Students completed a pre-survey to assess both knowledge and comfort levels regarding discharge summaries. They wrote a discharge summary and received feedback from two evaluators, an inpatient provider (IPP) familiar with the described patient and a simulated primary care provider (PCP). Students completed a post-survey reassessing knowledge and comfort.

**Results**: Prior to instituting this curriculum, the majority of students (92.9%) reported rarely or never receiving feedback on discharge summaries. Eighty-four of 88 (95.5%) eligible students participated. There was discordance between IPP and simulated PCP feedback on their assessment of the quality of discharge summaries; simulated PCPs gave significantly lower global quality ratings (7.9 versus 8.5 out of 10, p = 0.006). Key elements were missing from >10% of discharge summaries. Student response was favorable.

**Conclusion**: Clinically relevant deficiencies were common in students’ discharge summaries, highlighting the need for earlier, structured training. IPPs and simulated PCPs gave discordant feedback, emphasizing differing needs of different providers during transitions of care. This novel curriculum improved students’ knowledge and confidence.

## Introduction

Hospital discharge represents a vulnerable and potentially dangerous transition period. As many as one-third of patients experience an adverse event after discharge, and Medicare claims data shows nearly one in five patients are readmitted [1,2]. Improved communication between inpatient and outpatient providers allows for smoother transitions of care and helps to ameliorate risk. Timely, high-quality discharge summaries enhance communication between providers and may ultimately improve patient outcomes. A transitions of care policy statement by the American College of Physicians, Society of General Internal Medicine, Society of Hospital Medicine and others emphasizes the importance of timely and accurate information exchange during transitions of care [3].

In hospitals across the USA, advanced practice providers (APPs) are an integral part of inpatient care. A recent study suggests that greater than 50% of inpatient services utilize APPs, and data from 2017 indicates over 40% of graduating physician assistant (PA) students will work in the inpatient setting [4,5]. Thus, PAs will be increasingly responsible for discharging patients and authoring discharge summaries. Despite their expanding role as inpatient providers, we are not aware of any published curricula to train PAs, PA residents, or PA students to write effective discharge summaries.

In physician education, there is limited literature describing efforts to train medical students and/or house staff to write discharge summaries. In our previous work, we found that discharge summaries prepared by fourth-year medical students demonstrated frequent errors and omissions, emphasizing the need for early, standardized education [6]. Published curricula have been feasible to implement and improved trainees’ competence; however, programs have struggled with sustainability and delivery of personalized feedback [6–9].

In order to address the lack of standardized curricula and in response to the limitations of previously described interventions, we developed and implemented a curriculum at our institution to teach second-year PA students to write effective discharge summaries. The aim of this report is to describe the design, implementation, and outcomes of this novel curriculum. We utilized a unique dual feedback system compromised of an inpatient provider (IPP) familiar with the described patient and a faculty advisor with no role in the patient’s care to simulate a receiving primary care provider (PCP). We hypothesized that this curriculum would teach students not only to write an accurate synopsis of a patients’ hospital course but also to develop discharge summaries that effectively facilitate transitions of care.

## Methods

### Curriculum structure

Our institution is a large PA program affiliated with an academic medical center in North Carolina with approximately 90 students enrolled per class. All second-year students participated in this curriculum during the 2016–2017 academic year. Prior to their clinical experiences, students attended a lecture on discharge summary documentation given by one of the authors of this study (DM). On their required internal medicine (IM) rotation, students rotated at two clinical sites, including institution-affiliated hospitals and a variety of community locations. A quarter of the students did not rotate through an institution-affiliated hospital and, due to non-integrated electronic medical records (EMRs) and concerns regarding the Health Insurance Portability and Accountability Act (HIPAA), were unable to complete the standard curriculum. These students participated in an alternate ‘review and reflect’ pathway as described below.

The curricular intervention consisted of four steps. First, students completed a pre-survey, assessing knowledge of and comfort with writing discharge summaries. Students then wrote a discharge summary on a patient for whom they cared during their inpatient IM rotation. Students were encouraged, but not required, to use the standard discharge summary template created by our institution’s hospital medicine faculty that was available within our EMR. The discharge summary was de-identified and submitted for feedback to two evaluators, a supervising IPP and a PA program faculty advisor. IPPs were most often the inpatient attending physician of service, but could also have been supervising PAs or senior resident physicians who cared for the described patient. Advisors, meant to represent simulated PCPs, were one of four senior faculty who had not cared for the described patient. Advisors are assigned to students each academic year and serve in an advisory and evaluator role to their cohorts longitudinally. Students received feedback from each evaluator, then completed a post-survey.

As stated above, certain students could not complete *de novo* discharge summaries due to their clinical rotation site, i.e., completing their IM rotation at community programs with an EMR that was not integrated with our institution. These students participated in an alternate ‘review and reflect’ pathway. They were asked to review a previously authored discharge summary on a patient for whom they provided care. Students evaluated the discharge summary and indicated the presence or absence of key elements. They also wrote an essay critically appraising the discharge summary, responding to the following prompt: ‘Please write a reflection on your experience as the user and evaluator of this discharge summary. Highlight your thoughts about the summary’s quality/utility, and identify areas with opportunities for improvement. Consider what information you wish had been included in the DC summary, and/or whether the available information could have been communicated in a more user-friendly manner.’ Students submitted a de-identified copy of the discharge summary and essay to their advisor for review and feedback. They did not receive IPP feedback.

### Evaluations and surveys

**Figure 1.**
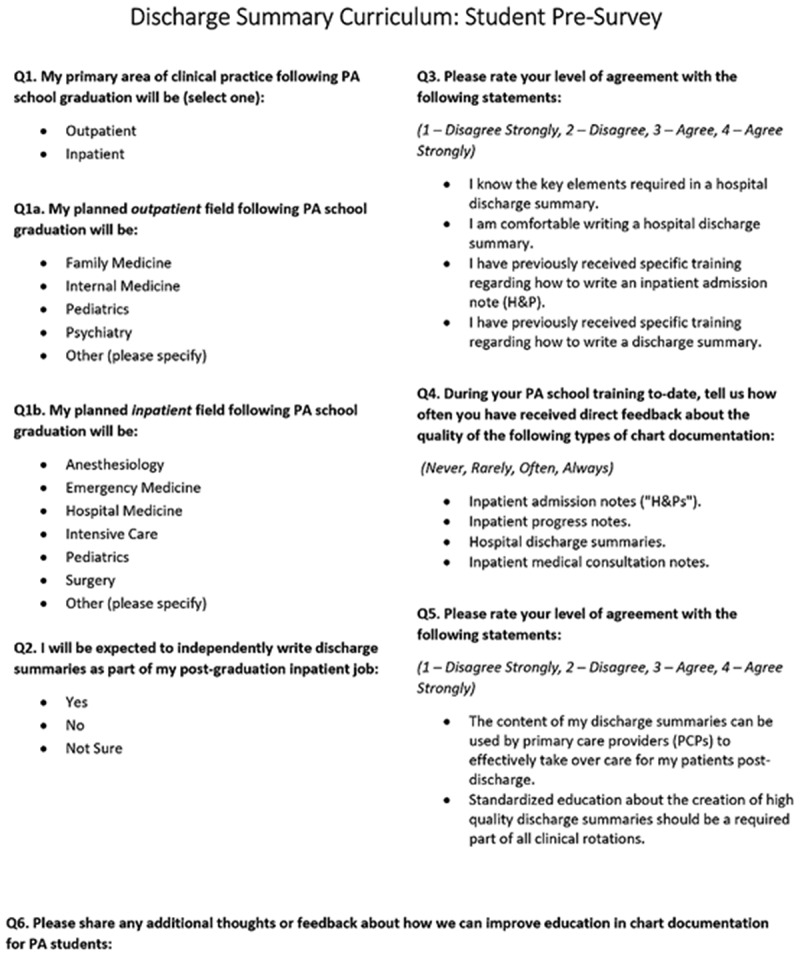
Students were asked to complete this pre-survey prior to independently authoring a discharge summary. They completed a similar post-survey after completion of the curriculum.

Student pre- and post-surveys consisted of four-level Likert-scale questions that queried knowledge and confidence in writing discharge summaries (Figure 1). The pre-survey collected information about students’ prior experience authoring and receiving feedback on clinical documentation (admission history and physicals (H&Ps), daily progress notes, discharge summaries). The post-survey also solicited open-ended feedback on students’ perceptions of the curriculum.

**Figure 2.**
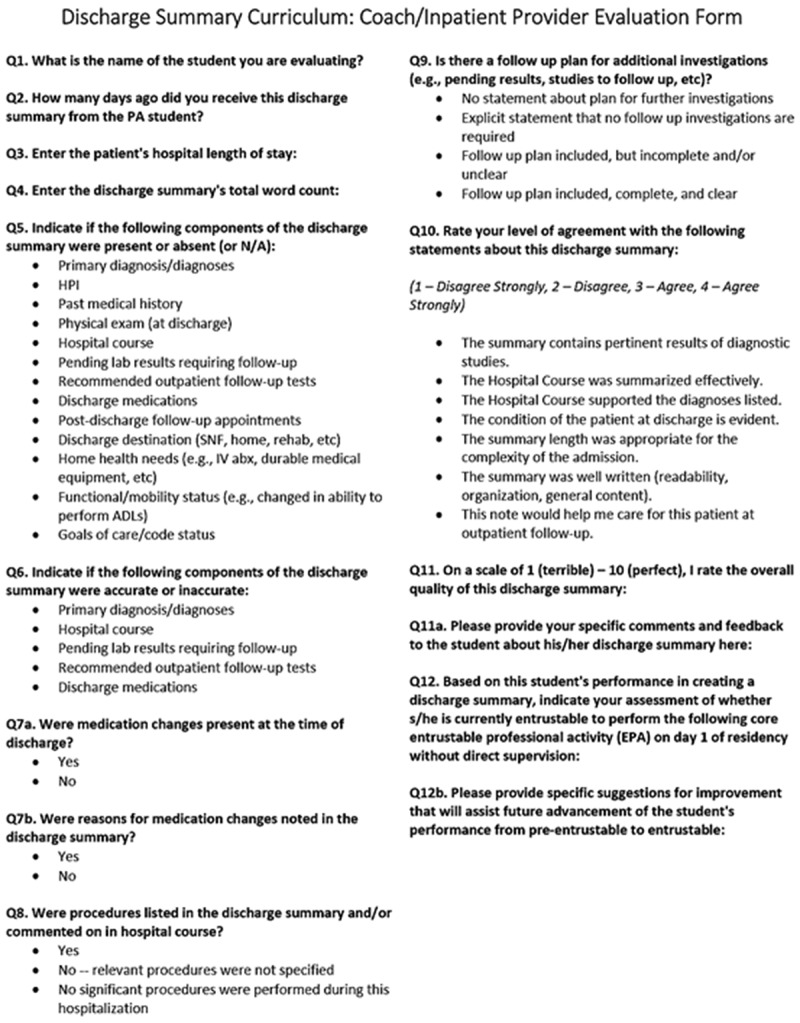
Evaluators rated submitted discharge summaries using this standardized template.

Advisors underwent formal training and evaluated four sample discharge summaries with the evaluation tool to help promote the standardization of scoring. Due to scheduling constraints and the large number of eligible providers, we did not offer formal training to IPPs. Evaluators rated students’ discharge summaries using a standardized evaluation tool created by one author (DM), which was based on previously described tools and used with permission (Figure 2) [9–11]. Evaluators rated the quality of the discharge summary by assessing for the presence or absence of key elements [12] and assigning a summative quality score for the discharge summary on a 10-point scale (1 = terrible, 10 = perfect). Evaluators also assessed whether students were entrustable to give or receive patient handover to transition care, as based on the American Association of Medical College guidelines for graduating medical students [13]. Evaluators utilized a similar form when evaluating review and reflect essays. We utilized an online survey platform (Qualtrics®) to automate the secure delivery of evaluations back to students. Students received feedback via Qualtrics on all components as shown in Figure 2, including information about the inclusion of key elements, global ratings, and free-text responses.

### Data analysis

We summarized student demographics, survey responses, evaluator response data, overall discharge summary quality, and entrustability with descriptive statistics. We compared advisors’ global ratings and entrustability of students who wrote *de novo* discharge summaries with those who completed the review and reflect pathway using an Exact Wilcoxon test and Fisher’s Exact test, respectively. We completed a match-paired analysis of IPP and advisor ratings for students who wrote *de novo* discharge summaries using a paired t-test to compare global ratings and McNemar’s test to compare entrustability, as well as the inclusion/exclusion of key elements. On the student pre-and post-survey, McNemar’s test was used to evaluate each matched statement. For analysis, agree/strongly agree and disagree/strongly disagree were grouped to create two levels. Tests for significance were two-sided using a significance level of α = 0.05; Bonferroni’s correction for multiple testing was used to test the inclusion of all key elements. Analyses were performed using SAS® version 9.4 (SAS Institute, Inc., Cary, NC). This intervention and analysis plan was reviewed by our IRB and met criteria for IRB exemption.

## Results

### Curriculum feasibility

**Figure 3.**
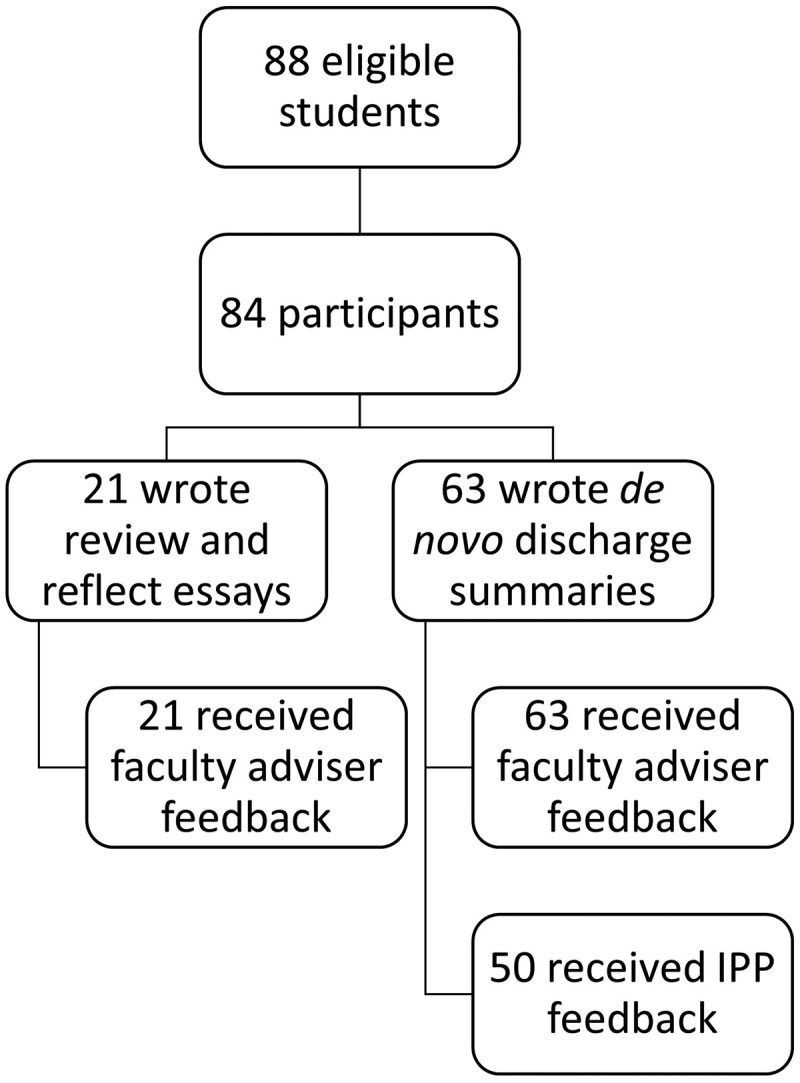
Demonstrates rates of student participation in discharge summary curriculum. Of 88 eligible students, 84 participated either via the review and reflect pathway (n = 21) or writing *de novo* discharge summaries (n = 63). All participating students received faculty advisor feedback. Of the 63 students who wrote *de novo* discharge summaries, 50 received additional feedback from inpatient providers (IPPs).

Eighty-eight students were in the class cohort, and of those, 84 (95.5%) completed at least some portion of the project (Figure 3). Sixty-three students (75.0%) wrote *de novo* discharge summaries and 21 (25.0%) wrote review and reflect essays; all 84 students received advisor feedback. Fifty of the 63 (79.4%) students who wrote *de novo* discharge summaries also received feedback from IPPs. Advisors and IPPs required a median of 11.8 (Interquartile range (IQR) 6.8–24.6) and 8.0 (IQR 5.9–11.4) minutes, respectively, to complete evaluations.

### Faculty evaluations

Twenty-one students participated in the review and reflect pathway. Twenty were rated as entrustable by advisors, with one missing response. Of the 63 students who wrote *de novo* discharge summaries, 57 (91.9%) were rated as entrustable by advisors and 5 (8.1%) as pre-entrustable with one missing response; the proportion of students rated as entrustable was similar between those who wrote *de novo* discharge summaries and those who completed the review and reflect pathway (p = 0.24). Students who participated in the *de novo* pathway had higher mean global quality scores, as rated by advisors (7.8 versus 6.4, p = 0.01).

Of students who wrote *de novo* discharge summaries, 50 received evaluations from both advisors and IPPs. Of these students, four (8.0%) were rated pre-entrustable by IPPs and 3 (6.1%) by advisors (p = 0.7). Advisors assigned lower global quality ratings on average than IPPs (mean 7.9 versus 8.5, p = 0.006). Advisors were also more likely to indicate key elements of discharge summaries as missing (Table 1), although differences did not reach significance. There was poor concordance of ratings of missing elements between IPPs and advisors, meaning elements rated as missing by IPPs were rated as present by advisors and vice versa. Past medical history, discharge medications, follow-up appointments, discharge destination, home health needs, functional status, and goals of care were each missing in greater than 10% of discharge summaries, as rated by either IPPs or advisors.

**Table 1.** Comparison of IPPs’ and advisors’ evaluations of new discharge summaries written by students.

Rating instrument	IPP	Advisor	Either
Overall quality, Mean (SD)	8.52 (1.09)	7.90 (1.43)*	
Percent entrustable by EPA, N (%)**	46 (92.00%)	46 (93.88%)	
**Missing rates of key elements**, N (%)			
Primary diagnoses	0 (0%)	0 (0%)	0 (0%)
History of present illness	1 (2%)	0 (0%)	1 (2%)
Past medical history	2 (4%)	5 (10%)	7 (14%)
Physical exam at discharge	2 (4%)	1 (2%)	3 (6%)
Hospital course	0 (0%)	0 (0%)	0 (0%)
Pending lab results	2 (4%)	0 (0%)	2 (4%)
Outpatient follow-up tests	3 (6%)	1 (2%)	4 (8%)
Discharge medications	0 (0%)	0 (0%)	0 (0%)
Follow-up appointment	1 (2%)	5 (10%)	6 (12%)
Discharge destination	2 (4%)	6 (12%)	7 (14%)
Home health needs	3 (6%)	9 (18%)	12 (24%)
Functional/mobility status	4 (8%)	7 (14%)	10 (20%)
Goal of care/code status	4 (8%)	6 (12%)	7 (14%)

The top portion of the table indicates how *de novo* discharge summaries were rated on an overall global scale (1–10, with 10 being perfect) and the number of students rated as entrustable to receive patient care handoff by inpatient providers (IPPs) and faculty advisors. The bottom portion of the table indicates whether key elements were rated as missing by IPPs and advisors. There was poor concordance among evaluators, so the third column indicates if an element was rated as missing by either evaluator. An item is listed as present if it was listed in the discharge summary or appropriately listed as ‘not applicable’ (e.g., explicitly indicated no follow-up tests are needed would count as ‘present’ under the item outpatient follow-up tests). *Global rating scales were significantly higher by IPPs than faculty advisors (p = 0.006). **There were 50 IPP entrustability ratings from IPPs and 49 entrustability ratings from faculty advisors, with one missing faculty advisor response.

### Student evaluations

**Figure 4.**
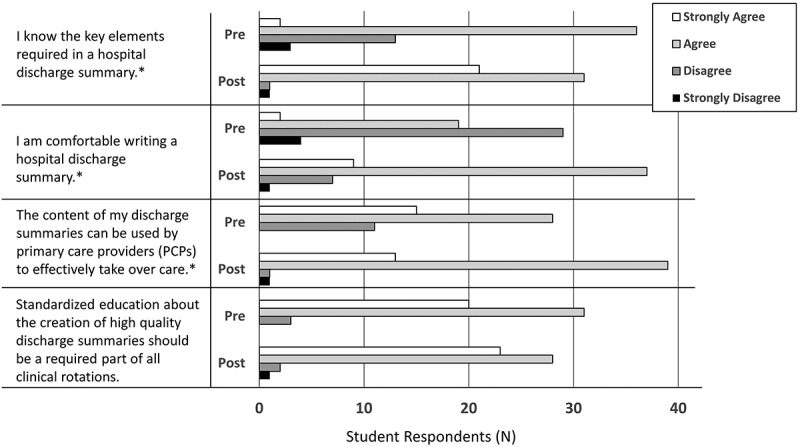
Demonstrates student responses (N = 54) to four matched statements on the pre- and post-surveys. *Combining agree/strongly agree and disagree/strongly disagree into two groups, students demonstrated significant improvement on knowledge of key elements (p < 0.001), comfort in writing a discharge summary (p < 0.001), and confidence that their discharge summaries could effectively be used to transition care (p = 0.011).

Eighty-five students responded to the pre-survey. Of those, 79 (92.9%) reported having rarely or never received feedback on discharge summaries when compared to 23 (27.1%) on H&Ps and 28 (32.9%) on progress notes. Fifty-four students responded to both the pre- and post-surveys. Responses on four paired statements are shown in Figure 4. Combining agree/strongly agree and disagree/strongly disagree into two groups, students demonstrated significant improvement on knowledge of key elements (p < 0.001), comfort writing a discharge summary (p < 0.001), and confidence that their discharge summaries could be used to transition care (p = 0.01).

## Discussion

We implemented a novel curriculum to teach PA students how to write high-quality, effective discharge summaries. At our institution, prior to instituting this curriculum, over 90% of students reported never or rarely receiving feedback on discharge summary documentation. Nine students (10.7%) were rated as pre-entrustable to appropriately document transition of care from the inpatient to the outpatient setting by either advisors or IPPs and over 10% of discharge summaries were missing key elements despite the use of a pre-populated template in our EMR (Table 1). These deficits emphasize the need for structured intervention early in students’ training, as close to half of PA students will go on to inpatient practice and be expected to independently author discharge summaries [5].

Our curricular intervention had several strengths. This curriculum was feasible to implement, with 95% student participation rate. Faculty evaluations required a median completion time of 8–12 min, and we utilized an automated, online system so students received timely feedback. Feedback from students was positive overall, with students reporting significant improvements in their perceived knowledge and confidence regarding discharge summary documentation. The curriculum was integrated into a required IM clerkship during which students are expected to author H&Ps and progress notes and receive feedback from supervising providers. Thus, the discharge summary exercise tied in well with pre-existing clerkship requisites. We also believe that writing discharge summaries based on real-world patient care, rather than simulated patients, improved student engagement. Another major strength was the dual feedback system. Students received feedback from both an IPP and an advisor simulating a receiving PCP. Advisors assigned lower global ratings than IPPs and there was poor concordance on entrustability ratings and inclusions of key elements between evaluators. These contrasting perspectives and incongruent feedback highlight the fact that discharge summaries must meet the needs of multiple providers. Discharge summaries serve not only as narrative synopses but also as tools to facilitate the transition of care between providers.

### Limitations

We conducted a single-center study at a large PA program, which may limit the generalizability of results. Students were expected to submit a single discharge summary for feedback during their rotation; thus, we were unable to determine whether students’ documentation improved due to the intervention. In future iterations, we will ask students to write and submit a second discharge summary as an opportunity to incorporate the feedback they received on the first. IPP participation rates were lower than those of advisors, possibly due to decreased awareness of the project or time constraints while rounding on inpatient services. By raising awareness of the curriculum, we hope to continue improving IPP evaluation rates to a goal of 100% completion. IPPs were also unable to undergo formal training on the use of the evaluation tool; therefore, IPP ratings may be prone to greater variability when compared to advisors. Furthermore, senior internal medicine residents were eligible to provide feedback, and they themselves may not have received formal training on authoring discharge summaries, further contributing to variability [6]. In the future, limiting participating IPPs to supervisory attending physicians and PAs, as well as providing standardized training for IPPs, will help to standardize feedback, although there are many logistical barriers to overcome. Finally, while necessary to provide a meaningful experience for students rotating at non-affiliated hospitals, we feel the review and reflect pathway lacked the experiential process of authoring a unique discharge summary. We plan to eliminate the review and reflect pathway in future iterations of the curriculum, as we believe the process of writing optimizes learning. Written comments on the student post-survey indicated that students preferred writing *de novo* discharge summaries as well.

### Conclusions

In conclusion, we implemented a novel curriculum to teach second-year PA students to write high-quality, effective discharge summaries. Utilizing automated feedback delivery and dual feedback perspectives, students obtained experience and confidence in writing discharge summaries that can be used to transition care. Hospital discharge represents a high-risk transition of care for patients, with associated high rates of readmissions and adverse events [1,2]. High-quality discharge summaries can mitigate this risk through improved provider communication. Although PAs are taking on an expanding role as inpatient providers, we are not aware of any previously described curricula to teach students or residents to practice this essential skill [4,5]. Thus, we believe this curriculum helps to fill an important gap in PA education.
